# Nitric oxide induces MUC5AC mucin in respiratory epithelial cells through PKC and ERK dependent pathways

**DOI:** 10.1186/1465-9921-8-28

**Published:** 2007-03-29

**Authors:** Jeong Sup Song, Chun Mi Kang, Moon Bin Yoo, Seung Joon Kim, Hyung Kyu Yoon, Young Kyoon Kim, Kwan Hyung Kim, Hwa Sik Moon, Sung Hak Park

**Affiliations:** 1 Department of Internal Medicine, ST Mary's hospital, Catholic University Medical College. #62, Yeoi-Do Dong, Young Dung Po Gu, Seoul, Korea

## Abstract

**Background:**

Nitric oxide (NO) is generally increased during inflammatory airway diseases. This increased NO stimulates the secretion of mucin from the goblet cell and submucosal glands but the mechanism is still unknown precisely. In this study, we investigated potential signaling pathways involving protein kinase C (PKC) and mitogen-activated protein kinase (MAPK) in the NO-induced MUC5AC mucin gene and protein expression in A549 cells.

**Methods:**

Nitric oxide was donated to the A549 cells by NOR-1. MUC5AC mucin levels were assayed by enzyme-linked immunosorbent assay (ELISA). MUC5AC promoter activity was determined by measuring luciferase activity after the lysing the transfected cells. Activation of PKC isoforms were measured by assessing the distribution of the enzyme between cytosolic and membrane fractions using immunoblotting. Immunoblotting experiments using a monoclonal antibody specific to PKC isoforms were performed in the cytosol and membrane fractions from A549 cells. Western blot analysis for pERK and p38 were performed using the corresponding antibodies from the cell lysates after donating NO to the A549 cells by NOR-1.

**Results:**

The transcriptional activity of MUC5AC promoter was maximal at the concentration of 0.1 mM NOR-1 for 1 hour incubation in transfected A549 cells. (±)-(E)-methyl-2-((E)-hydroxyimino)-5-nitro-6-methoxy-3-hexenamide (NOR-1) markedly displaced the protein kinase C (PKC)α and PKCδ from the cytosol to the membrane. Furthermore, the PKC-α,βinhibitors, GÖ6976 (10 nM) and PKCδ inhibitors, rottlerin (4 μM) inhibited the NOR-1 induced migration of PKCα and PKCδ respectively. NOR-1 also markedly increased the MUC5AC promoter activity and mRNA expression, mucin synthesis and ERK1/2 phosphorylation. The PKC inhibitors also inhibited the NOR-1 induced MUC5AC mRNA and MUC5AC protein synthesis by inhibiting the activation of PKCα and PKCδ with ERK1/2 pathways.

**Conclusion:**

Exogenous NO induced the MUC5AC mucin gene and protein through the PKCα and PKCδ – ERK pathways in A549 cells. Inhibition of PKC attenuated NO-mediated MUC5AC mucin synthesis. In view of this findings, PKC inhibitors might be useful in the treatment of bronchial asthma and chronic bronchitis patients where NO and mucus are increased in the bronchial airways.

## Background

Production of NO is generally increased during inflammatory airway diseases such as asthma or bronchiectasis, or after exposure to irritant gases such as ozone [1]. NO is produced by the action of NO synthase (NOS) on L-arginine and has many physiological and pathological roles. In chronic lower airway disease, the role of NO include pulmonary vasodilation, brochodilation, regulation of ciliary beat frequency and mucus production [2,3] and NOS is found in raised quantities in the airway epithelium of asthmatic patients[4].

Goblet cell hyperplasia and metaplasia are well established hallmarks of the airways of cigarette smokers, with and without chronic obstructive pulmonary disease (COPD). Enhanced epithelial mucin expression is believed to be the rate limiting step for goblet cell metaplasia [5]. Four gel forming mucins (MUC2, MUC5AC, MUC5B, and MUC19) are found in the lung. Of these, MUC5AC and MUC5B are the major respiratory mucins present in secretions from goblet cells and sub-mucosal glands, respectively [6]. MUC5AC has been shown to be stimulated by a wide variety of stimuli, including pro-inflammatory cytokines such as IL-9, IL-1β and tumor necrosis factor (TNF)-α [7,8], neutrophil elastase [9], epidermal growth factor receptor (EGFR) ligands [10], air pollutants [11] and bacterial products [12]. Oxidants in cigarette smoke and generated from asbestos fibers activate mitogen-activated protein kinase (MAPK) signalling cascades in lung epithelial cells [13]. Airway MUC5AC mucin is transcriptionally upregulated by cigarette smoke and is mediated by an AP-1 containing response element binding JunD and Fra-1 [14]. Furthermore, it is reported that PKC is involved in TNF-α or bacterial components induced MUC2 and MUC5AC overexpression in airway and middle ear epithelial cells or goblet cells [15].

NO donation by isosorbide dinitrate increased MUC5AC mucin secretion in the goblet cell line HT29-MTX [16] but suppressed chemokine production in keratinocytes [17]. There have been only a few studies investigating the role of NO in airway mucus secretion and much is still unknown about the role of PKC and MAPK pathways during upregulation of MUC5AC mucin secretion after donation of NO to the bronchial epithelial cells. In this study, we evaluated the effect of NO release on MUC5AC mucin production and the cell-signaling pathways involved in its regulation in the cell line A549. A549, a lung adenocarcinoma cell line, which has been used extensively as a model of respiratory epithelium and expresses both MUC5AC mRNA and glycoprotein [18].

In this study, we examined effects of NO on MUC5AC mucin synthesis and PKC-mediated second messenger pathways that may be involved in physiological functions of airway epithelium. Our results suggest that the PKC inhibitors inhibit the MUC5AC mRNA expression and mucin synthesis through inhibiting the PKCα and PKCδ-ERK1/2-MUC5AC promoter pathways during donation of NO to the A549 cells.

## Materials and methods

### Cell culture

Human lung adenocarcinoma-derived A549 cells were cultured in Roswell Park Memorial Institute (RPMI1640) media supplemented with 10% fetal bovine serum (FBS), penicillin 100 U/ml and streptomycin 100μg/ml. Cells were maintained in a humidified incubator at 37°C with 95% air (vol/vol) and 5% (vol/vol) CO_2_. The cells were replenished with fresh media every 2–3 days. The cell viability was periodically determined by trypan blue exclusion method.

### Agonists and inhibitors

NOR-1 (Calbiochem, Darmstadt, Germany) was used as a NO donor. For control experiment, N^G^-nitro-L-arginine methyl ester (L-NAME) was used as a nitric oxide synthase inhibitor. Phorbol 12-myristate 13-acetate (PMA) was used as a protein kinase C (PKC) activator and inhibitors of PKC isoforms were used such as GÖ6976 (PKCα/β inhibitor), rottlerin (PKCδ inhibitor) and calphostin C (a ubiquitous PKC inhibitor) which were purchased from Calbiochem (Darmstadt, Germany).

### MUC5AC protein measurement by ELISA

MUC5AC protein was measured as described previously [19]. Briefly, 50 μl of A549 cell lysate and 50 μl of 2 × carbonate/bicarbonate buffer were loaded into the 96-well ELISA plates and dried at 44°C. The plates were washed three times with phosphate buffered saline (PBS) and blocked with 2% bovine serum albumin (BSA) for 1 h at room temperature. Then, it was incubated with 50 μl of mouse anti-human MUC5AC Ab (1:100 Neomarker, Fremont, CA) for 1 h. Plates were washed as above. Mucin detection was accomplished by addition of 100 μl/well of a 1:2,500 dilution of peroxidase-conjugated goat anti-mouse IgG in PBS containing 15% FBS and incubation for 1 h. Plates were washed as above. Colorimetric reaction was developed with 100 μl/well peroxidase substrate. Optical density (OD) measurements were obtained from an ELISA reader (BIO-TEK Instruments, Winooski, VT) at 405 nm, with 450 nm serving as the reference wavelength. Results were calculated by dividing the OD reading for mucin during the experimental period by the OD reading for the L-NAME-treated baseline mucin. Results were expressed as percent of baseline control.

### Measurement of nitrate and nitrite contents by Greiss assay

Nitrate and nitrite were measured via the Greiss assay in the culture media. 1 × 10^5 ^of A549 cells were seeded on 100 mm dish and incubated until 80–90% confluency. After adapted in serum-free medium for 24 h, cells were stimulated by NOR-1 for 3 h and supernatant was collected for Greiss assay. For Nitrate, 200 μl of culture media and 200 μl of nitrate reductase buffer that contained 50 μM NADPH, 40 mM KH_2_PO_4 _and 50 mU nitrate reductase were mixed and incubated at room temperature for 2 h. 200 μl of 0.8% N-1-naphthyl-ethylene diamine was added to same amounts of 2% sulfanilamide in 0.2 N HCl. After incubation at room temperature for 10 minutes, the absorbance was measured on a spectrophotometry at 540 nm. Nitrite of cell supernatant was determined using a mixture of 50 μl of 2% sulfanilamide in 0.2 N HCl and 50 μl of 0.8% N-1-naphthyl-ethylene diamine. Sodium nitrite was used as the standard.

### Transient Transfection

In size of 1.3 Kb fragment MUC5AC promoter which was cloned into the pGL3-Basic luciferase vector was generously provided by Carol Basbaum (University of California, San Fransisco). A549 cells were seeded on 6-well plates (2 × 10^5^cells/well) and incubated for 48 h in serum free medium. Before transfection, the pGL3-MUC5AC-3752pro luciferase reporter plasmid and control pGL3-Basic vector were adjusted to 200 ng/μl, and β-galactosidase was adjusted to 100 ng/μl. The tube designated 'A' contained 300 μl of serum media, 5 μl of pGL3-MUC5AC-3752pro luciferase reporter plasmid, 5 μl of Plus reagent (GIBCO BRL), and 3 μl of β-galactosidase, while 'B' tube contained 300 μl of serum free media and 4 μl of LIPOFECTAMINEβ REAGENT (GIBCO BRL). Each tube was mixed well in room temperature and 200 μl of the mixture was added to the wells containing A549 cells. After 5h, 1 ml of 20% FBS was added to the wells and further incubated for 24 h.

### Luciferase assay

In order to investigate the dose-dependency of NO on the MUC5AC promoter transcriptional activity, A549 cells were stimulated with 0.1, 0.5, 1 and 1.5 mM of NOR-1 for 1h. To examine the time-dependency, A549 cells were incubated with 0.1 mM of NOR-1 for 30 min, 1, 3, 5 and 24 h or PKC inhibitors for 30 min. MUC5AC promoter activity was determined by measuring luciferase activity after the lysing the transfected cells and normalizing by co-transfection with the β-galactosidase expression plasmid, pβ-gal control vector (Clontech). β-galactosidase activity was measured in the luminometer (Turner Designs, San Jose, CA) in accordance with the manufacturer's instructions. All transfections were performed in triplicate wells; results were reported as emitted light per well (mean ± SD).

### RT-PCR

Total RNA was isolated using TRIzol^® ^reagent (guanidium isothiocyanate-phenol mixture; Invitrogen, Charlsbad, CA) and chloroform from A549 cells. The RNA was incubated with 10 mM dNTP, 0.1 M DTT, 1 μl random hexamer (1 pmole) and 1 μl SuperScript II (200 U/μl Invitrogen, Charlsbad, CA) at 42°C for 50 min, and then heat-inactivated at 70°C for 15 min. After reverse transcription, PCR was performed with specific primer pairs for the MUC5AC and β-actin genes in a thermocycler (Bio-Rad, Hercules, CA) with an initial denaturation step of 94°C for 4 min, followed by 28 cycles of 1 min at 94°C, 1 min at 60°C, 1 min at 72°C, with a final extension at 72°C for 7 min. The following primer pairs were used for the PCR: MUC5AC, 5-TCC GGC CTC ATC TTC TCC-3 (forward) and 5-ACT TGG GCA CTG GTG CTG-3 (reverse); β-actin, 5-CAA GAG ATG GCC ACG GCT GCT TCC-3 (forward) and 5-TCC TTC TGC ATC CTG TCG GCA ATG-3 (reverse). The amplified PCR products were visualized on a 1% agarose gel by ethidium bromide staining.

### Separation of cytosol and membrane fractions and analysis of PKC isoforms

A549 cells (1 × 10^5^) were seeded on 100 mm dishes and cultured in 10 ml until 80–90% confluency. After PKC inhibitors were treated for 30 min, cells were washed and incubated with NOR-1 for 3 h. Cells were harvested by centrifugation (1,000 rpm, 5 min) and pumped by 1 ml syringe for destruction. For cytosol and membrane fraction, destroyed cells were centrifuged at 50,000 rpm (200,500 g, rotor type 100Ti, Beckman Coulter, CA, USA) for 1 h at 4°C, and then supernatant (cytosol fraction) was collected. After RIPA buffer (20 mM Tris-HCl, pH 7.4, 137 mM NaCl, 1 % Nonidet P-40, 0.25 % sodium deoxycholate, 0.1 % SDS, 1 mM EDTA, 10 ug/ml aprotinin, 1 mM PMSF, 0.1 mM sodium vanadate and 10 mM sodium fluoride) was added into the pellet (membrane fraction), it was sonicated about 5 s. Both fractions were quantitated by Bradford method and equal amount of protein (20 §P) were resolved separately on 7.5% of SDS polyacrylamide gradient gels and transferred to polyvinylidene difluoride (PVDF) membrane. After blocking, membranes were incubated with anti-PKC antibodies (PKC sampler kit, BD Biosciences, CA, USA) followed by horseradish peroxidase (HRP)-conjugated antibodies. The detection was performed using a chemiluminescence method (Amersham Life Science). The density of signals was quantified using a densitometer.

### Western blot for MAPK

Cultured A549 cells were washed 3 times with cold PBS. After detached from the plates using scrapping, the cells were harvested by centrifugation (12,000 rpm for 20 minutes, 4°C). Cells were destroyed by RIPA buffer on ice for 20 minutes. After destroyed cells were centrifuged, proteins were collected from supernatant and determined by Bradford method. 50 §P of protein were separated on a discontinuous 10 % and 4% PAGE gel and then the proteins were transferred to a PVDF membrane at 80 V for 1 h. The membrane was blocked with 5 % skim milk in TBS buffer (10 mM Tris-Hcl, 150 mM NaCl, pH 7.5) for 1 h, and then incubated with the mouse anti-human p-ERK antibody (1:1000 Santa Cruz Biotechnology, Santa Cruz, CA) or rabbit anti-human p-p38MAPK antibody (1:1000 Cell signaling, Danvers, MA) at 4°C overnight. The membrane was washed 3 times with TBST buffer (TBS + 0.1% Tween20) and incubated with HRP-conjugated secondary antibody (1:2000) at room temperature for 1 h. The target protein was detected by ECL Kit (Amersham Pharmacia Biotech, Little Chalfont, Buckinghamshire, UK) using X-ray film.

### Statistical analysis

All data are presented as means ± SE. Data obtained from all the experiments was analyzed by Kruskal-Wallis one-way non-parametric analysis of variance with post hoc evaluations by Mann-Whitney's rank sum test (SAS Institute, Cary, NC). A level of significance was considered at p < 0.05.

## Results

### NO concentration in A549 cells culture media

**Figure 1:**
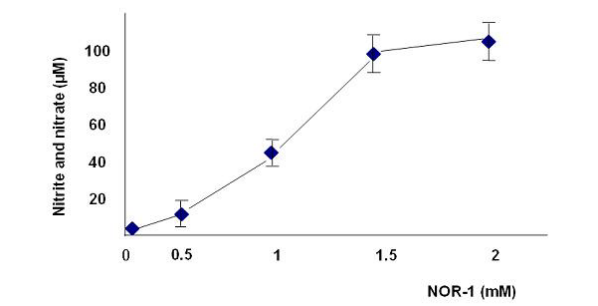
Effects of the NO donor, NOR-1 on nitric oxide secretion from the A549 cells. The nitrite and nitrate concentrations were measured at 540 nm by Griess reagent method after stimulation with different concentrations of NOR-1 for 3 hours.

The concentrations of NO in the culture medium of A549 cells after incubation with the synthetic NO donors, NOR-1 for 3 hours were well correlated the concentrations of NOR-1 (Fig. 1). The NO concentrations in the culture medium were quantified by measuring nitrite and nitrate concentrations using the Greiss reaction [20].

### Effect of NO donation on MUC5AC promoter activity

**Figure 2:**
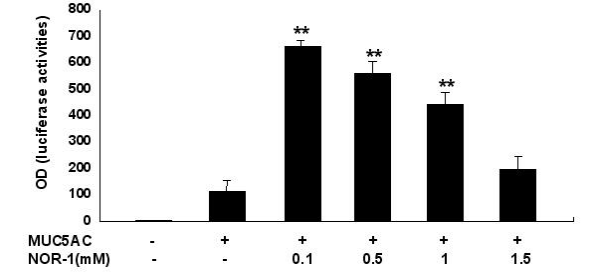
NOR-1 increased the transcriptional activity of MUC5AC promoter. A549 cells were transfected with MUC5AC promoter. The transfected cells were treated with vehicle or different concentrations of NOR-1 for 1 hr and then harvested for measurement of luciferase activities. ** significantly different, p < 0.01, from MUC5AC promoter-alone transfection group.

**Figure 3:**
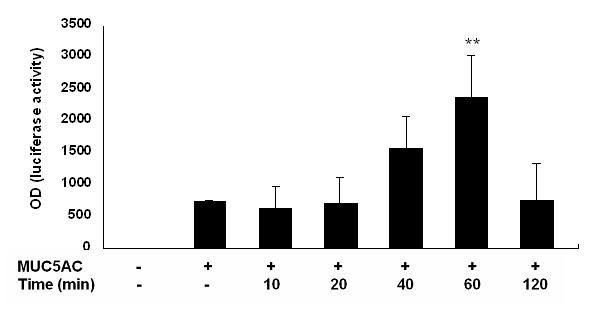
Time course of the effect of NOR-1 on MUC5AC promoter activity. A549 cells were transfected with vehicle or MUC5AC promoter. Transfected cells were stimulated with 0.1 mM of NOR-1 and the transcriptional activity of MUC5AC promoter was measured at 10, 20, 40, 60 and 120 min. after exposure. ** significantly different, p < 0.01, from MUC5AC promoter-alone transfection group.

To determine whether NO was regulating MUC5AC transcription, we transfected A549 cells with a luciferase reporter pGL3-basic vector containing the 3.7 kb 5' flanking region from the transcription start site of the human MUC5AC promoter. NOR-1 increased the transcriptional activity of MUC5AC promoter most markedly at the concentration of 0.1 mM (Figure 2) and 60 minute incubation (Figure 3). MUC5AC transcriptional activity was increased after stimulation with NOR-1 for one hour between 0.1 mM and 1 mM concentrations (Figure 2).

### Activation of PKC isoforms by NOR-1

**Figure 4:**
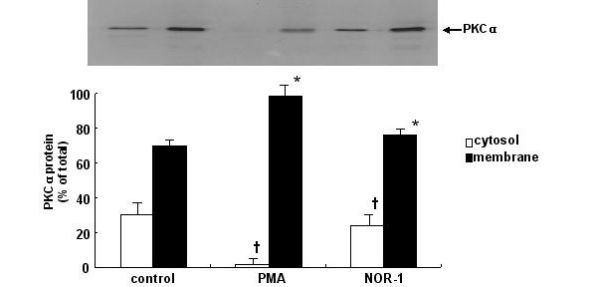
Effects of NO donor and PMA on the distribution of PKCα in A549 cells. A549 cells were exposed to NOR-1 (0.5 mM) or PMA (1 μM) for one hour and then fractionated. Proteins of equal amounts were separated by SDS-PAGE, transferred, incubated with anti-PKCα antibodies, and detected using a chemiluminescence method. The results were expressed as means ± SE of three independent experiments. * p < 0.01 versus control membrane. † p < 0.01 versus control cytosol.

**Figure 5:**
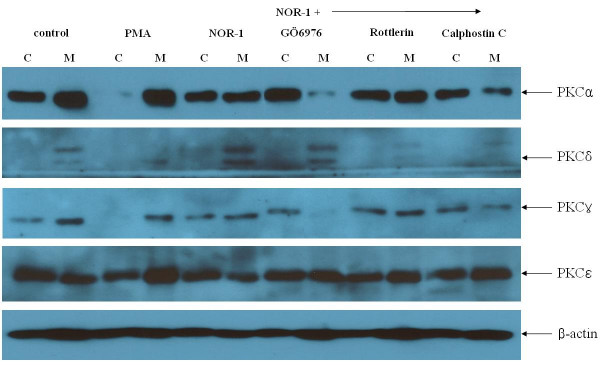
Effects of NOR-1 on PKC isoforms expression in A549 cells. Cell extracts were portioned into cytosol (C) and membrane (M) fractions as described under "Materials and Methods." PKC isoforms were detected by Western blotting. NOR-1 (0.5 mM) induced migration of PKCα and PKCδ but not PKCγ and PKCε from the cytosol to the membrane. PKC-α,β inhibitors, GÖ6976 (10 nM) and PKCδ inhibitors, rottlerin (4 μM) inhibited the NOR-1 induced migration of PKCα and PKCδ respectively. NOR-1 induced migration of PKCα and PKCδ were also inhibited by calphostin C (0.5 μM).

To confirm the role of PKC activation in the effect of NO on MUC5AC mucin synthesis in A549 cells, we assessed the effects of NOR-1 on PKCα. Activation of PKCα was measured by assessing the distribution of the enzyme between cytosolic and membrane fractions using immunoblotting, because translocation of the enzyme from the cytosolic fraction to the membrane fraction correlates with activation of the enzyme. As shown in Figure 4, incubation with NOR-1 for one hour resulted in significant translocation of PKCα from the cytosolic fraction to membrane fraction. The translocation of PKCα was more prominent during incubation with 1 μM phorbol 12-myristate 13-acetate (PMA), a PKC activator. Next, we tested the effect of NOR-1 on PKC isoforms expression in A549 cells. As shown in figure 5, 0.5 mM NOR-1 induced migration of PKCα and PKCδ from the cytosol to the membrane. The coincubation with PKCα,βinhibitors, GÖ6976 (10 nM) and PKCδ inhibitors, rottlerin (4 μM) inhibited the NOR-1 induced migration of PKCα and PKCδ respectively. NOR-1 induced migration of PKCα and PKCδ were also inhibited by 0.5 uM calphostin C, a general PKC inhibitor.

### Effect of NOR-1 and PKC inhibitors on mucin secretion

**Figure 6:**
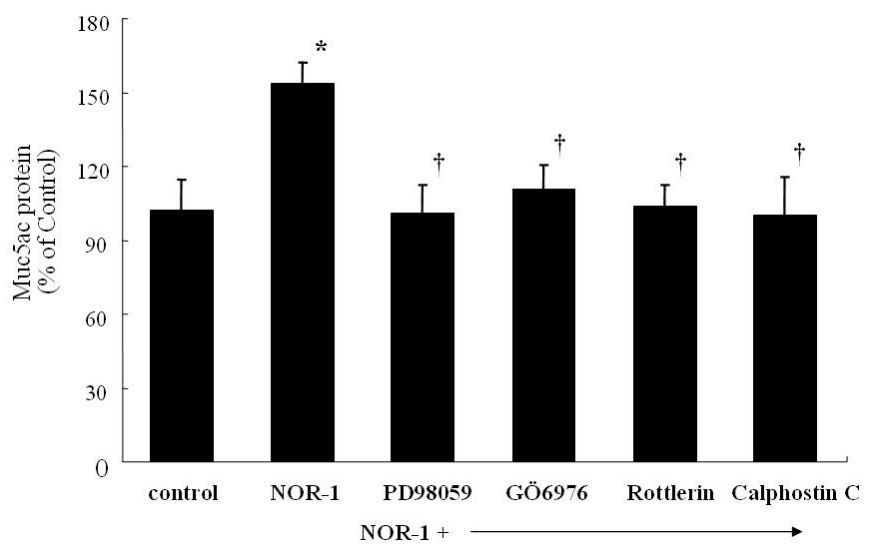
Effects of NOR-1 and PKC inhibitors on the MUC5AC mucin synthesis from the A549 cells. A549 cells were exposed to NOR-1 (0.5 mM) in the presence of ERK-inhibitor, PD98059 (40 μM) or PKC-α,β inhibitors, GÖ6976 (10 nM) or PKC-δ inhibitors, rottlerin (4 μM) or specific PKC inhibitors, Calphostin C (0.5 μM). The results were expressed as means ± SE of eight different experiments. * p < 0.05 versus control, † p < 0.05 versus NOR-1 stimulated cells.

As illustrated in Figure 6, NOR-1 stimulated MUC5AC mucin synthesis by A549 cells. The increased mucin synthesis elicited by the NOR-1 was reversed with the preincubation with GÖ6976, rottlerin and calphostin-C. No cytotoxic effects were observed.

### NOR-1 phosphorylated ERK1/2 but not P38 MAPK

**Figure 7:**
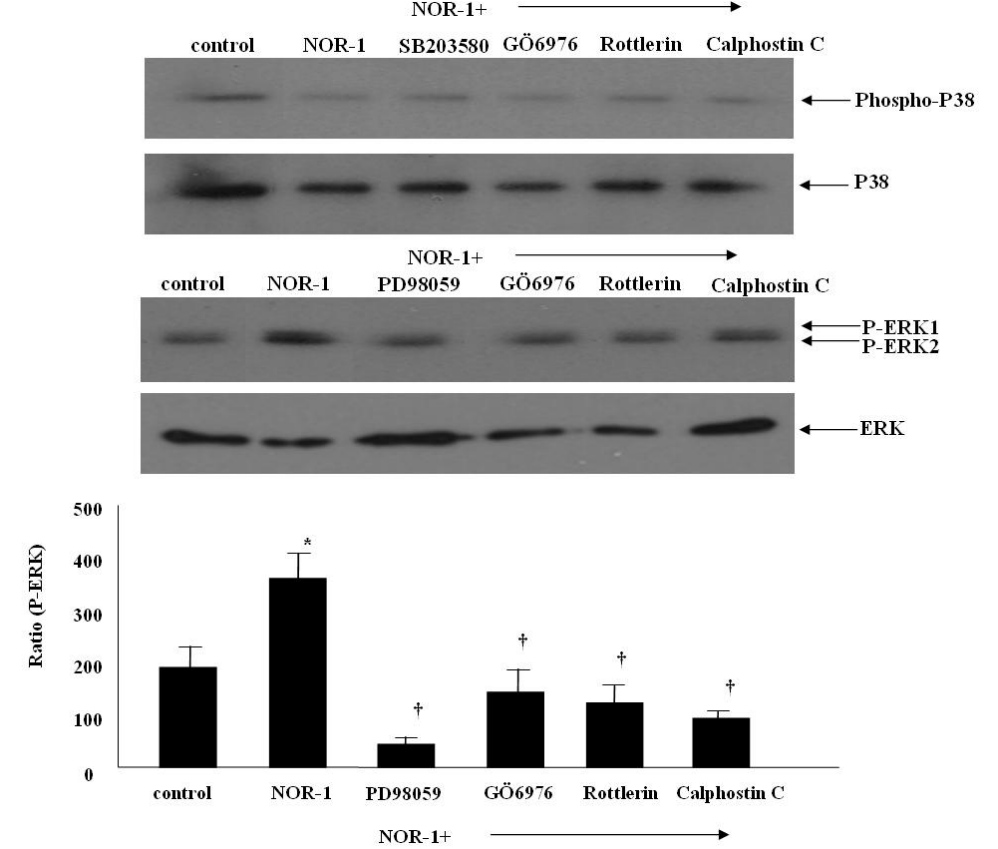
Effects of NOR-1 and PKC inhibitors on the expression of phosphorylated p38 and ERK1/2 protein in A549 cells. NOR-1 phosphorylated ERK1/2 but not p38 and PKC inhibitors, GÖ6976 (10 nM), rottlerin (4 μM), and Calphostin C (0.5 μM) inhibited the ERK1/2 phosphorylation.

As illustrated in Figure 7, exposure of A549 cells to NOR-1 caused a phosphorylation of ERK1/2 and this increased phosphorylation was inhibited with PD98059 (a specific MEK inhibitor), and PKC inhibitors (GÖ6976, rottlerin and calphostin C). However, the effects of NOR-1 on P38 MAPK phosphorylation was not noted.

### Effect of NOR-1 and PKC inhibitors on MUC5AC mRNA expression

**Figure 8:**
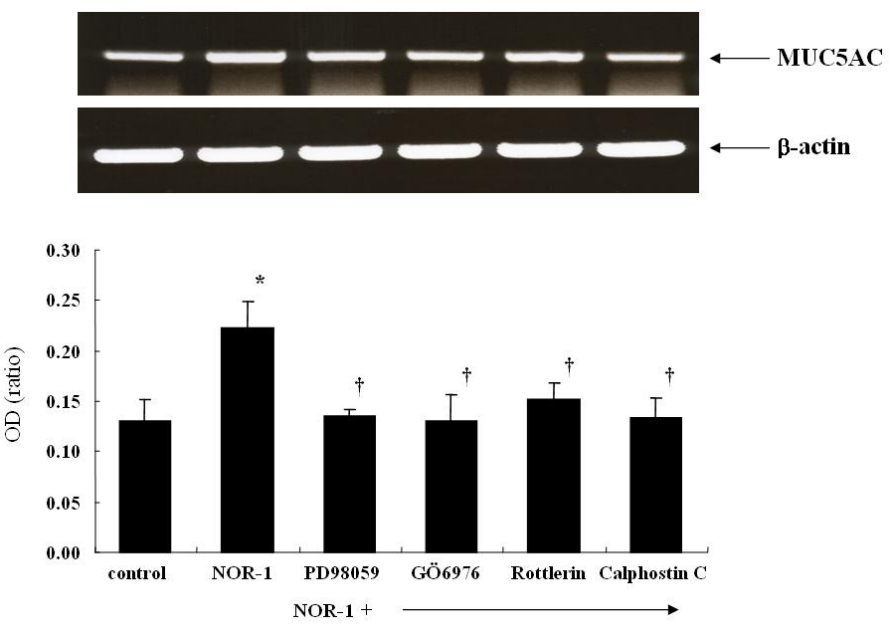
RT-PCR analysis of MUC5AC mRNA expression from A549 cells. Total RNA was extracted from confluent cultures and analyzed for the presence of MUC5AC and GAPDH transcripts by RT-PCR. The amplified products were run on 1% agarose-ethidium bromide gels. The results were expressed as means ± SE of six different experiments. * p < 0.01 versus control, † < 0.05 versus NOR-1 stimulated cells.

NOR-1 increased the MUC5AC mRNA expression and the PKC inhibitors (GÖ6976, rottlerin and calphostin C) inhibited NOR-1 induced MUC5AC mRNA expression (Figure 8).

## Discussion

The present study clearly demonstrates a potent stimulatory effects of NO donor on MUC5AC mucin secretion from A549 cells. Activation of the PKCα and PKCδ with ERK1/2 mediated NO donor induced MUC5AC mucin gene expression and mucin synthesis. We used NOR-1 as a NO donor which releases NO with a more rapid kinetics [21]. NO donors suppress chemokine production by inhibiting nuclear factor-kB and STAT-1 [22]. The role of NO in the regulation of inflammatory responses has been extensively investigated. However, there have been only a few studies investigating the role of NO in mucus secretion with conflicting results. On the one hand, NO inhibited mucus secretion in ferret trachea in vitro [23] and on the other hand, it had a stimulatory role in the mucus secretion in isolated submucosal gland from feline trachea [24] or it had no effect on the mucus secretion in the rat trachea [25].

Protein kinase C (PKC) is a family of serine/threonine-specific protein kinases with at least 10 different isoforms [21]. The PKC family contains three types of isoforms; classical (cPKCs: α, β_1_, β_2_, γ), novel (nPKCs: δ, ε, η, θ, μ), and atypical (aPKCs: ξ, ι/λ). The classical isoforms are calcium and phorbol ester-activated, the novel are calcium-insensitive but activated by phorbol esters, and the atypical isoforms are both calcium and phorbol ester-insensitive, with all isofoms activated by phosphatidyl serine[26].

The interaction between NO and PKC has been the subject of many studies, with most focused on the role of PKC in the regulation of NO production [27,28]. With regard to effects of NO on PKC, controversial results exist. NO inactivates PKC in a macrophage cell line [29]. On the other hand, NO activates PKC in hepatocytes [30], smooth muscle cells [31], and kidney cells [32]. In addition, NO was shown to mediate the stimulation of phospholipase C (PLC), a typical upstream step for PKC activation, by oxidant stress [33]. In a lot of inflammatory airway diseases, tumor necrosis factor (TNF)-α is involved in bronchoconstriction, pulmonary edema, and production of cytokines and lipid mediators. TNF-α stimulates mucin secretion via an intracellular pathway that appears to involve endogenously produced NO [34]. NO mediates many of its intracellular effects through activation of soluble guanyl cyclase with subsequent increased cyclic guanosine monophosphate (cGMP) production [35]. Recently NO has also been demonstrated in goblet cells to upregulate MUC5AC production [16].

In this study, NOR-1 directly increased the transcriptional activity of transfected MUC5AC promoter, indicating that NO-induced upregulation of MUC5AC mRNA occurs at the transcriptional level. NOR-1 also moved the PKCα and PKCδ from the cytosol to the membrane and this intracellular activation of PKC was inhibited by PKCα inhibitor and PKCδ inhibitor.

Involvement of PKC in secretion of airway mucin in response to various stimuli has been indicated previously [35-38]. The specific PKC isoenzymes that contribute to PKC-induced mucin secretion have not been determined, although PKCξ and PKCδ have been suggested as potential candidates [36,38,39]. Recently human neutrophil elastase has been found to induce mucin secretion through a PKCδ-mediated mechanism in human bronchial epithelial cells [40]. In this paper, we also found that the MUC5AC mucin synthesis by NOR-1 was inhibited by PKC inhibitors. As illustrated in figure 8, NOR-1 increased the MUC5AC mRNA expression and this increased expression was nearly completely inhibited by PKC inhibitors. The calphostin C; a specific PKC inhibitor, rottlerin; a PKCδ/θ inhibitor, GÖ6976; a PKCα/β inhibitor all inhibited the NOR-1 induced MUC5AC mRNA expression, MUC5AC mucin synthesis and extracellular signal-regulated kinases (ERKs) phosphorylations. Calphostin C is a specific PKC inhibitor that binds to the diacylglycerol (DAG) binding site of the enzyme to block its activity [41]. Our findings suggested that NO activated both α and δ forms of PKC which in turn involved in MUC5AC mucin synthesis in A549 cells. When we examined the translocation of PKC isoforms in response to NOR-1, NOR-1 activated the PKCα and PKCδ but not PKCγ and PKCε (figure 5). As expected, the activation of PKCα by NOR-1 was inhibited by GÖ6976 and the activation of PKCδ by NOR-1 was inhibited by rottlerin. Calphostin C inhibited the NOR-1 induced activation of both PKCα and PKCδ.

Phorbol esters, such as phorbol 12-myristate 13-acetate (PMA), are important inflammatory stimuli that have been shown to modulate diverse cellular events through PKC activation [42]. PMA induced an increase in MUC2 gene expression and this induction involved PKC, was Ras and Raf dependent, required activation of mitogen-activated protein/ERK kinase (MEK) and extracellular regulated kinase (ERK) pathways, and led to the activation of the cis-acting transcription factor, NF-kB [43]. MUC5AC mucin was also induced by PMA through the Ras-Raf-MEK/ERK and specificity protein (Sp) 1 transcription factor dependent pathways [44].

The mitogen-activated protein kinase (MAPK) cascades consist of serine threonine kinases that are sequentially phosphorylated by upstream kinases (MAPKKK, MAPKK) and subdivided into three major pathways: ERKs, c-Jun-NH_2_-terminal kinases (JNKs 1, 2, and 3) (also referred to as stress-activated protein kinases), and p38 kinases [45,46]. MAPK cascades can be initiated by activation of receptor tyrosine kinases such as the epidermal growth factor receptor (EGFR) or other factors stimulating phosphorylation of upstream MAPKKK and MAPKK (MEK). Oxidative stress causes activation of EGFR-MEK-ERK1/2 pathways, resulting in mucin synthesis [47]. Recent studies have demonstrated cross-talk between p38 MAP kinase and ERK [48,49]. p38 MAP kinases are activated by a variety of agents, including environmental stress (e.g., reactive oxygen species, UV radiation), cytokines (e.g., interleukin [IL]-1β, tumor necrosis factor [TNF]-α), or growth factors such as EGF and platelet-derived growth factor (PDGF) [50,51].

In this study, we found that NO donation by NOR-1 activated ERK1/2 but not p38 and this ERK1/2 activation was inhibited by several types of PKC inhibitors and by MEK inhibitor, PD98059 (figure 7). These findings suggest that NO induced MUC5AC mucin through the PKC-MEK-ERK1/2 pathways in A549 cells. According to previous reports on respiratory tract and colon epithelial cells, production of mucin induced by gram-positive or gram-negative bacteria is dependent on tyrosine kinase such as the MEK1/2-MAPK signalling pathway [52-55]. This tyrosine kinase signal results in the activation of NF-kB in respiratory tract epithelial cells, which are involved in the overproduction of mucin induced by Psudomonas aeruginosa [54].

Today, it is widely accepted that NO plays an important role in airway function. NO is an important mediator in the lung and has been shown to be associated with inflammatory lung diseases such as asthma and chronic bronchitis [56-58]. In addition, overproduction of mucus with altered rheologic properties is an important factor in the morbidity and mortality of asthma and chronic bronchitis [59,60]. Our results suggest that PKC inhibitors may be a promising new agents for the treatment of mucin hypersecretion in inflammatory airway diseases where NO is highly produced.

## Abbreviations

NOR1 = (±)-(E)-methyl-2-((E)-hydroxyimino)-5-nitro-6-methoxy-3-hexenamide; NO = nitric oxide; PKC = protein kinase C; ELISA = enzyme linked immunosorbent assay; TNF-α = tumor necrosis factor α; EGFR = epidermal growth factor receptor; ERK = extracellular signal-regulated kinase; PMA = Phorbol 12-myristate 13-acetate
